# Chemical Composition, Antioxidant and Antibacterial Activities and Acute Toxicity of *Cedrus atlantica*, *Chenopodium ambrosioides* and *Eucalyptus camaldulensis* Essential Oils

**DOI:** 10.3390/molecules28072974

**Published:** 2023-03-27

**Authors:** Rabab Ez-Zriouli, Houda ElYacoubi, Hamada Imtara, Abdelhalim Mesfioui, Aboubaker ElHessni, Omkulthom Al Kamaly, Samar Zuhair Alshawwa, Fahd A. Nasr, Zineb Benziane Ouaritini, Atmane Rochdi

**Affiliations:** 1 Laboratory of Natural Resources and Sustainable Development, Research Unit of Agro-Physiology, Biotechnology and Environment, Faculty of Sciences, Ibn Tofail University, Kenitra 14000, Morocco; 2 Faculty of Sciences, Arab American University Palestine, Jenin 44862, Palestine; 3 Biology and Health Laboratory, Faculty of Sciences, Ibn Tofail University, Kenitra 14000, Morocco; 4 Department of Pharmaceutical Sciences, College of Pharmacy, Princess Nourah bint Abdulrahman University, P.O Box 84428, Riyadh 11671, Saudi Arabia; 5 Department of Pharmacognosy, College of Pharmacy, King Saud University, Riyadh 11451, Saudi Arabia; 6 Laboratory of Biotechnology and Natural Conservation of the Resources, Dhar El Mehraz Faculty of Science, Sidi Mohamed Ben Abdellah University, Fes 30000, Morocco

**Keywords:** essential oils, secondary metabolites, microbial resistance, cytotoxicity

## Abstract

The essential oils yield of *Cedrus atlantica*, *Chenopodium ambrosioides* and *Eucalyptus camaldulensis* was different. *C. ambrosioides* gave a relatively higher yield (2.1 ± 0.1%), while that of *C. atlantica* was low (1.0 ± 0.1%) and that of *E. camaldulensis* was lower (0.75 ± 0.1% of dry matter). The active ingredients of the essential oils and some of their biological effects were also determined. The characterization of their chemical compositions showed that the three essences have different chemical profiles: *C. atlantica* was richer in sesquiterpenes (β-Himachalene (54.21%) and γ -Himachalene (15.54%)), *C. ambrosioides* was very rich in monoterpene peroxides and monoterpenes (α-Terpinene (53.4%), ascaridole (17.7%) and ρ-Cymene (12.1%)) and *E. camaldulensis* was very rich in monoterpene compounds and monoterpenols (p-cymene (35.11%), γ-Eudesmol (11.9%), L-linalool (11.51%) and piperitone (10.28%)). The in vitro measurement of antioxidant activity by the 2,2-diphenyl-1-picrylhydrazyl radical (DPPH) reduction assay showed a significant performance of the eucalyptus oil and average performance of the other two (*C. atlantica* and *C. ambrosioides)*. The in vitro bio-test for their antimicrobial effects showed that the antibacterial activity differed depending on the essential oil and the concentration used, and that their bactericidal efficacy was similar or superior to that of synthetic antibiotics. The toxicity test on rats revealed that the LD_50_ of the three essential oils was 500 mg/kg body weight, which classifies them as category four cytotoxic natural products at high doses.

## 1. Introduction

Today, the impact of used synthetic antibiotics on human health is a concern [1]. The increase in bacterial resistance to antibiotics has prompted industries to seek alternative strategies based on natural sources such as essential oils (EOs) and plant extracts [2,3]. The antiseptic benefits of aromatic and medicinal plants and their extracts have been recognized since antiquity, while attempts to characterize these properties in the laboratory date back to the early 1900s [4].

A lot of antibacterial infections can be effectively treated with aromatic and medicinal herbs [5], since they have no negative effects and contain a variety of secondary metabolites [6]. Moreover, herbal medicines continue to be the mainstay of therapy for major ailments in developing nations. Indeed, traditional remedies are still used to treat common illnesses for between 60 and 80 percent of the world’s population [7]. However, recent research has shown that antioxidants, which are used to combat free radicals, can also be utilized to treat some pathologies, including diabetes, cancer and neurodegenerative diseases. In contrast, many illnesses, including carcinogenicity, can be brought on by the usage of synthetic antioxidants [5,8].

Due to safety issues related to the use of synthetic compounds, EOs extracted from aromatic and medicinal plants have received more attention as a natural product with many applications in different fields, including food preservation, cosmetics, perfume industry, pharmacy and food processing [9]. In this sense, research must be applied to the study of new plants in order to examine their chemical profiles, as well as their biological and pharmacological power.

*Chenopodium Ambrosioides* (M’khinza in Moroccan) is a wild species from tropical America naturalized in the old world [10,11]. It is an annual perennial species that grows in moist and low soils [12] and its form varies; occasionally, it is also persistent. It does not exceed 1 m in height, its leaves are oval, lanceolate and coarsely toothed, the plant is covered with aromatic glandular hairs and its flowers are often reddish, grouped in small sessile glomerules and are tight in the last branches, with yellow, protruding anthers. The fruits contain very small, brownish, lenticular shiny seeds [13]. Several works have addressed the antileishmaniasis properties of this plant: antioxidant, anthelmintic, anti-inflammatory, antimicrobial and insecticide [14,15,16]. Traditionally, this plant has been used as a remedy for parasitic diseases [17] and to treat parasitosis because of the presence of ascaridol [17]. In Morocco, the plant is commonly used as an infusion or fresh juice in gastrointestinal diseases and dysentery [6]. It can also be used in terms of a local application of the fresh plant, in the case of oral abscesses, ulcerations and purulent wounds [6].

*Eucalyptus camaldulensis*, is native to mainland Australia [11]. It is part of the Myrtaceae family, and it includes 900 species and subspecies [12]. Today, eucalyptus is found almost everywhere in the world, as it is cultivated in many countries [13]. *Eucalyptus* are large trees, some of which can exceed 100 m in height, but the average of the most common species is 40–50 m; other trees are smaller in size [14]. The leaves are entire and leathery with a strong cutin; they are evergreen and aromatic. Members of the genus *Eucalyptus* are known to be important reservoirs of a wide range of secondary metabolites, many of which have several biological activities [15]. *Eucalyptus* leaves are a common antispasmodic and antipyretic remedy used to treat respiratory tract diseases [16]. The essential oils of eucalyptus have long been used within the field of pharmacy to make antiseptics, inhalants and embrocations [14].

*Cedrus atlantica* (Atlas cedar) is a species endemic to the mountains of North Africa. It is a coniferous tree that tolerates drought and cold and can grow on clay and loam soils. The tree can reach a height of 30 to 40 m, and is of the Pinaceae family [17]. Its longevity is very remarkable and it can exceed 1000 years. The Atlas cedar is a monoecious species. It develops light yellow male flowers and greenish female flowers and the inflorescences do not develop at the same time; pollination begins in October. The cone is 5 to 8 cm long, and it is green before maturity and then it is brown, and it matures in two years [18,19,20,21,22,23,24,25,26,27]. In Morocco, the Atlas cedar occupies an area of 132,000 hectares [28,29]. It is highly valued for its technological, ecological and biogeographical values [22]. It is highly sought after for its many uses (sawing and charcoal) [23]. The essential oils of *Cedrus atlantica* are known for their antifungal, antimicrobial, antiviral and anti-inflammatory properties [30,31,32]. The essential oil of *Cedrus atlantica* is used in the composition of many products, such as perfumes and medicines [33,34].

The objective of this study was to evaluate the antioxidant and antibacterial activity on human pathogenic strains and the acute oral toxicity of *Cedrus atlantica*, *Chenopodium ambrosioides* and *Eucalyptus camaldulensis* essential oils.

## 2. Results and Discussion

### 2.1. Yield and Chemical Composition

The average yields of essential oils were calculated on the dry matter of the relevant parts of the plant. *E. camaldulensis* essential oil provided a relatively low yield of about (0.75 ± 0.1%), this yield is relatively low when compared to that obtained from *E. camaldulensis* plants harvested from Antoniades garden in Alexandria and the north west region of Algeria (0.9%) [27]. Additionally, other researchers have found that the highest values of *E. camaldulensis* yield are in the range of 1.2% and 1.4% [28,29]. For the samples of *C. atlantica*, they provided a yield in the order of (1.0 ± 0.1%), which was lower than that of both the Boulmane region (1.82%) [30] and the Eastern Middle Atlas (1.12 ± 0.2%) [20]. On the other hand, this yield is higher than that obtained from the samples of this plant from the Bainem forest (0.005%) and the Aures region in Yabous (El Kantina) (0.017%) in Algeria. However, the *C. ambrosioides* species are provided with the highest yield with about (2.1 ± 0.1%), which is relatively higher compared to that obtained from *C. ambrosioides* from Benin, which varies between 0.3 and 1.2% [33,34]; additionally, to that obtained from plants collected in Akure metropolis, Nigeria (0.52%) [35]. The hydrodistillation of the aerial part of *C. atlantica* from Cameroon also gave a low yield of essential oil (0.12%) compared to our study [36].

These variations in EO yields are likely due to different environmental factors. Age and sampling season may also affect EO yield [37].

Chromatographic analysis of the essential oils identified 15 compounds representing about 100% for the essential of *E. camaldulensis*, 20 compounds (99.4%) for the essential oil of *C. ambrosioides* and 15 compounds (97.11%) for that of *C. atlantica* (Table 1).

The essential oil of *E. camaldulensis* is composed mainly by p-cymene (35.11%), γ-eudesmol (11.9%), L-linalool (11.51%) and piperitone (10.28%). On the other hand, Chaves et al. asserted that 1,8-cineole (76.93%), β-pinene (11.49%) and α-pinene (7.15%) are the main constituents of *E. camaldulensis* leaf essential oil collected in Brazil [38]. Some differences in the compositions were found by Gakuubi et al., for example the oil is rich in 1,8-cineole (16.2%), α-pinene (15.6%), α-phellandrene (10.0%) and p-cymene (8.1%) [11]. *E. camaldulensis* samples collected in Alexandria, Egypt, were rich in spathulenol (20.84%), eucalyptol (12.01%), sabinene (9.73%),α-phellandrene (8.18%) and crypton (7.69%) [39]. Other researchers have shown that the essence of *E. camaldulensis* is dominated by 1,8-cineole (42.30%) and α-pinene (28.30%) [29]. Studies conducted on species collected from the Antoniades garden in Alexandria gave different results with the dominance of eucalyptol (55.36%), α-pinene (14.87%), γ-terpinene (8.77%) and the (-)-terpinene-4-ol (5.23%) [27].

The essential oil of *C. atlantica* is jointly dominated by β-himachalene (54.21%) and γ -himachalene (15.54%). Other compounds are also present but at reduced levels: Himachalene oxide (6.23%) and Limonene (6.12%). All these compounds contribute to the mixture at 82.1%. This chemical composition was relatively similar to other analyzed *C. atlantica* essential oils [40] whose main compounds were Himachalene with a content of about 31.24%, followed by α-himachalene (15.63%) and γ-himachalene (14.46%). In addition, in other works, this oil is rich in α-himachalene (35.34%) followed by β-himachalene (13.62%), γ-himachalene (12.6%), cedrol (10.32%), isocedranol (5.52%) and α-pinene (5.5%) [20].

Compared to other results reported in the literature, the compounds found in the *C. atlantica* essential oil in high percentages in plant samples from Morocco are α-pinene (14.85%), himachalene (10.14%), β-himachalene (9.89%), σ-himachalene (7.62%) and cis-α-atlantone (6.78%) [30].

The samples of *C. ambrosioides* provided an essential oil rich in α-terpinene (53.4%), ascaridole (17.7%) and ρ-Cymene (12.1%) in addition to other constituents with relatively low contents: carvacrol (7.3%) and Isoascaridole (2.1%). These compositions are substantially similar to that studied by Hsu et al., where the essential oil of *C. ambrosioides* collected in northern Taiwan was found to be composed of α-terpinene (30.5%), p-cymene (17.3%), carvacrol (16.2%) and ascaridole (15.1%) [41]. The chemical composition of the *C. ambrosioides* essential oil from Rwanda was specific: it was low in ascaridole (7.2%) when compared to the oils described in the literature.

The essential oils analyzed by GC/MS in the present work showed different chemical profiles, whereby the essential oil of *E. camaldulensis* is very rich in monoterpene compounds and monoterpenols, and *C. atlantica* richer in sesquiterpenes. On the other hand, the essential oil of *C. ambrosioides* is very rich in monoterpenic peroxides and monoterpenes.

We emphasize that qualitative and quantitative changes in the chemical composition are largely related to several factors, including species, topographic conditions that characterize the regions and areas where plants material were collected, climatic (temperature and humidity) and edaphic conditions, plant age, genotype, maturity, harvesting period and procedure and extraction technique [6,42,43].

### 2.2. Antioxidant Activity

The essential oil samples evaluated were capable of reducing the stable violet radical of DPPH to DPPH-H yellow, and the concentration values differed depending on the oil evaluated and the control used. The antioxidant power of the EOs was represented as a Trolox equivalent (mg TE/g of EO), ascorbic acid equivalent (mg AAE/g EO) and hydroxytoluene (mg BHTE/g EO).

The antioxidant potential differs depending on each oil (Table 2). According to the results obtained, the *Eucalyptus* samples gave the best results: they were able to reduce the DPPH radical with the values of 99.252 mg BHTE/g EO, 24.167 mg AAE/g EO and 40.406 mg TE/g EO, which could be explained by the richness of this oil in secondary metabolites especially in hydroxylated compounds: L-linalool 11.51%, α-terpineol 2.67%, 1.8 cineol 1.44% and Linalool 0.96%. In this regard, the antioxidant activity of eucalyptus essential oils has been proven by other researchers [44,45]; these EOs have been reported to be able to be used to decrease diseases associated with oxidative stress, and also as food preservatives [46], [47]. The results obtained for *Cedrus atlantica* and *Chenopodium ambrosioides* samples are less important compared to previous researches; in fact, Skanderi et al. [23] reported that *Cedrus atlantica* essential oil exerted a remarkable antioxidant efficacy. Similarly, the EO of *Chenopodium ambrosioides* harvested in Guercif had a similarly high antioxidant activity [48]. The change in the antioxidant potential of essential oils can be attributed to the presence of certain molecules that have antioxidant activity [49]. Although the amounts of these compounds are relatively small in the oil, the possible synergistic and antagonistic effects of the compounds can also be considered [44].

### 2.3. Antibacterial Activity

The study of the antimicrobial activity of natural substances such as essential oils and synthetic products (e.g., antibiotics on microbial pathogens) is of great interest. In this respect, the antibacterial activity of commercialized antibiotics and essential oils of *Eucalyptus camaldulensis*, *Cedrus atlantica* and *Chenopodium ambrosioides* has been tested in vitro against human pathogenic strains.

**Figure 1:**
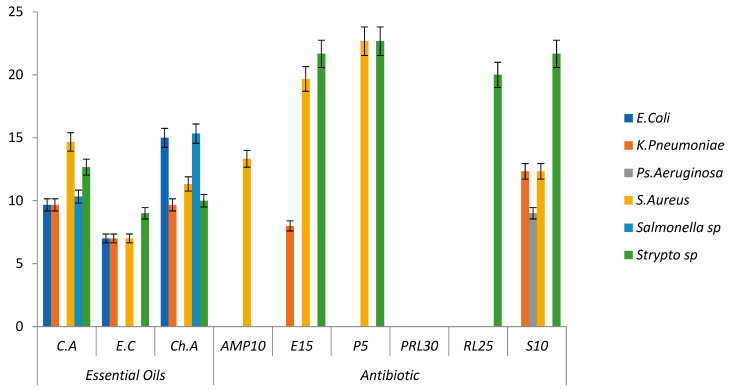
The effects of substances (essential oil and antibiotics) on the diameter of the inhibition zone. Data are presented as means (±SE).

The results of the disk diffusion method allowed us to establish an antibiogram (Figure 1). The results reveal an important activity, namely that the EOs reacted positively on most of the tested bacterial strains. The diameters of inhibition are close or superior to those recorded for antibiotics.

Analysis of variance (ANOVA) was used to evaluate the effect of species, substance (essential oil and antibiotic) and their interaction on the diameter of the zone of inhibition. The results show that the differences between strains, oils and antibiotics were very highly significant *p* < 0.0001 (Table 3). The microbial species did not react in the same way toward the essential oils and antibiotics.

The results of the antibacterial activity of *Eucalyptus camaldulensis* essential oil showed that the bacteria are moderately sensitive, except for *Salmonella* sp. and *Ps. aeruginosa*, which are the most resistant [50]. A rather broad inhibitory power of *eucalyptus* oil toward microorganisms was detected. In the same sense, previous studies have proven that the essential oil and extracts of *Eucalyptus camaldulensis* have a broad antimicrobial spectrum [51].

*Cedrus atlantica* EO inhibited the growth of some strains studied, even *E. coli*, which is resistant to all the antibiotics evaluated. *S. aureus* was found to be very sensitive, followed by *Streptococcus* sp. and *Salmonella* sp., while *Ps. aeruginosa* was the most resistant. Some authors have advanced the resistance of *Staphylococcus aureus* compared to other strains to essential oils of *C. Atlantica* [52]. Others have proven that strains react differently depending on the composition of cedar oil [53].

In the case of *Chenopodium ambrosioides* essential oil, the results showed that all the tested strains are sensitive to the effect of this essential oil, except *Ps. aeruginosa* (resistant strain). Moreover, the most pronounced effect was recorded against *Salmonella* sp. with an inhibition diameter of 15.33 ± 2.33 mm, noting that this strain is resistant to all the antibiotics evaluated. The antibacterial effect of *Chenopodium ambrosioides* essential oil is reported in several studies [54]. Bacteria *Bacillus subtilis* and *Escherichia coli* are resistant to antibiotics and have shown a promising sensitivity toward essential oils. Additionally, [55] reported in their studies that Gram-positive bacteria (*Bacillus cereus* and *Micrococcus luteus*) were generally more sensitive than Gram-negative bacteria.

Based on the results of the solid medium dilution method, the minimum inhibitory concentration (MIC) and minimum bactericidal concentration (MBC) values of EOs against the tested microorganisms are presented in Table 4. *Cedrus atlantica* essential oil showed potential antibacterial properties. *S. aureus* and *Klebsiella pneumonia* were the most susceptible since 1/2000 *v*/*v* of the extract exerted a bactericidal effect on these strains (MIC = MBC = 1/2000). While *Ps. Aeruginosa* appeared resistant even at the highest concentration of EO (1/100 *v*/*v*). Previous works indicated that *E. coli*, *Ps. aeroginosa* and *S. aureus* were the most sensitive strains to *Cedrus atlantica* oil [30].

*Chenopodium ambrosioides* oil was bactericidal against *K. pneumoniae* and *Salmonella* sp. at 1/500 and *E. coli* at 1/1000 *v*/*v*, but inhibited the growth of other strains, not including *Ps. aeruginosa*, which showed resistance. Other studies have confirmed the inhibitory action of essential oils of *Chenopodium ambrosioides* against *S. aureus* than on *E. coli*. Nevertheless, these tested essential oils showed bactericidal activity on *E. coli* with a minimum bactericidal concentration between 25.75 ± 1.80 and 27.45 ± 1.37 mg/mL and bacteriostatic activity against *S. aureus* [33].

*Eucalyptus Camaldulensis* essential oil exerted a bactericidal effect on *strypto* sp. (MIC = MBC = 1/250 *v*/*v*). In relation to other strains, the MIC was not determined; the concentration must be increased to have inhibition. In addition and based on other studies, at a 1/500 *v*/*v* concentration of *Eucalyptus Camaldulensis* oil in the culture medium, *E. coli* and *S. aureus* are more sensitive to *Eucalyptus Camaldulensis essential* oil [56].

The sensitivity of bacteria is independent of the Gram, it varied according to the germ tested. Thus, an essential oil can exert a bactericidal effect on some strains, and bacteriostatic on others, demonstrating the mutagenic character of these strains that allows them to develop resistance to antibiotics [57].

Interestingly, the bacteria with the largest zones of inhibition are not always those with the lowest MICs [58]. This may be due to the fact that the diameter of the zone of inhibition is affected by the volatility of the EO and its solubility in water [59].

The antimicrobial activity of EOs differs depending on the oil and the dilutions used. The total chemical composition is taken into account, The activity could well be due to the presence of a synergy between the main components tested and other constituents of the oils with varying degrees of antimicrobial activity [60]. On the other hand, some studies have shown that the antimicrobial activity of EOs is higher than that of its major compounds when tested separately [61]. The dominance of the activity of essences over that of a majority component confirms the synergistic effect that minority components could bring to the activity of EOs [62].

The antimicrobial efficacy of *Chenopodium Ambrosioides* may be due to its chemical profile, which is rich in monoterpenes (α-Terpinene 53.4% and p-Cymene 12.1%). These compounds possess strong antimicrobial activities; they exert their antimicrobial activity on microorganisms by disrupting the membrane integrity of bacteria [63], [64], resulting in the inhibition of respiration and the alteration of cell permeability [63].

The himachalene fractions present in the essence of *Cedrus atlantica* are already known by their insecticidal activity [65] and could have antimicrobial effects as well. Some authors have suggested the resistance of *S. aureus* to other strains of *Cedrus atlantica* essential oils [52]. Others have shown that strains react differently depending on the composition of cedar oil [53]. There is some evidence that minor components have a critical role to play in antibacterial activity, perhaps by producing a synergistic effect between other components [66].

The antimicrobial spectrum of eucalyptus essential oil and extracts toward microorganisms has been proven in previous research [58,59]. This inhibition could be attributed to the presence of antimicrobial substances in their chemical profile, as well as the interactive effect between them; the functional groups of some compounds present in most plant materials, alcohol, phenols, terpenes and ketones, are associated for their antimicrobial characteristics [56].

The tests of acute oral toxicity were carried out in order to determine the lethal dose 50 (LD_50_) of all the oils studied. The results obtained represented in Table 5.

The results of the acute toxicity study of the three plants, *Eucalyptus camaldulensis*, *Cedrus ctlantica* and *Chenopodium ambrosioides*, respectively, by the oral route, showed a number of deaths depending on the dose and the oil used as well as clinical signs of toxicity after administration.

For *Chenopodium ambrosioides* oil, from the dose of 300 mg/kg of body weight, only one mortality was observed during 14 days, and the rats of the other batches all died after administration of the 1000 and 2000 mg/kg doses. For *Eucalyptus camaldulensis* and *Cedrus ctlantica* oils, one and three deaths were recorded, respectively, from the 1000 mg/kg and 2000 mg/kg body weight doses. The severity of the toxicity increases with the increase in the dose. The confirmatory dose on new healthy rats is conducted and it led to similar results to the previous ones.

Based on these results, the LD_50_ was 500 mg/kg. According to the Globally Harmonized System of Classification and Labeling of Chemicals GHS, the three oils can be classified into category four.

The parameters determined during the observation period for the duration of 14 days showed that at the dose 300 mg/kg, the rats treated with the essential oil of *Chenopodium ambrosioides* manifested signs of sedation; their movements were reduced during the first hours after injection but they did not present a staggering gait or contractions.

We also noticed that from the 1000 mg/kg dose onwards, all the animals that received the *Eucalyptus camaldulensis* and *Cedrus ctlantica* oils assumed a retreating position, with their head tilted, eyes closed and breathing accelerated. However, a return to a normal state (activity and nutrition) was observed in some, while others maintained the weakened state until death.

The weight of the rats was monitored at the same time each day. A decrease in body weight can be explained by a reduced food consumption following the ingestion of the toxicant and the sensitivity of the animal to the extract.

**Figure 2:**
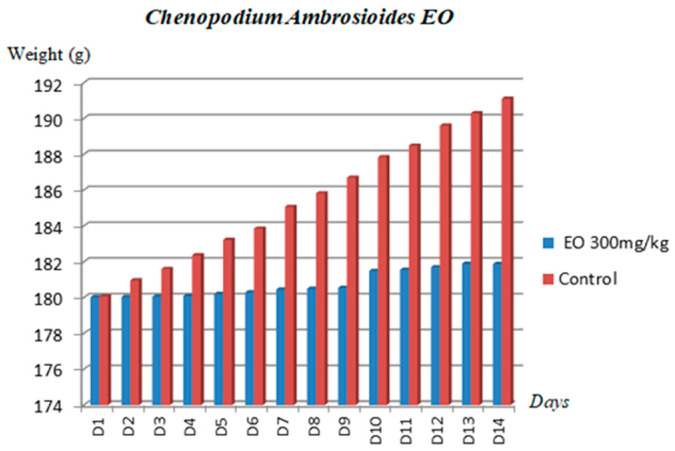

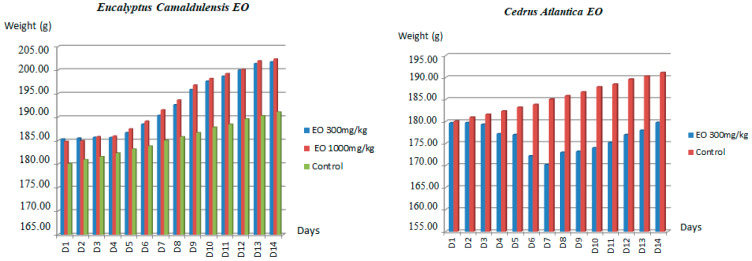
Variation in body weight of rats after administration of *E.C*, *Ch.A* and *C.A* oils during 14 days.

The follow-up of the weight evolution of the tested rats during the 14 days showed that the body weight underwent a regression from the first day of observation, and then when the signs of toxicity started to disappear, the weight progressively increased and exceeded the initial weight (Figure 2).

The in vivo toxicity test using the animal model by measuring the toxicity by body weight of the animals showed cytotoxic effects depending on the concentration and the oil. In the case of *Chenopodium ambrosioides* oil, studies showed that the LD_50_ was 131.63 μL/Kg [67], which is lower than what was found in our study. The LD_50_ of *Cedrus atlantica* oil was higher than 50 mg/kg [68]. The toxic effect could be attributed to the administration method and chemical composition of the extract. Studies by Monzote et al. (2009) [69] attributed the cytotoxicity of two chemotypes of *Cedrus atlantica* essential oil to carvacrol and caryophyllene. In addition, ascaridole was found to be toxic to HaCaT cells [6]. The lack of bibliographic data does not allow us to explain with certainty the toxic effect that our samples present at the doses used, but we assume that the majority of compounds in each EO are responsible for the signs of toxicity observed.

## 3. Materials and Methods

### 3.1. Plant Material

Fresh plants of *Cedrus atlantica*, *Chenopodium ambrosioides* and *Eucalyptus aamaldulensis* were collected during the flowering period (May–June) from different regions of Morocco (Table 6); the parts of each plant collected were dried for three weeks in the shade in an airy place and at room temperature in order to preserve the integrity of their molecules. Thus, the plants became dry, and they were stored in clean bags until the moment of extraction.

### 3.2. Extraction of Essential Oils

The extraction of essential oils was carried out by hydrodistillation with a Clevenger type apparatus [70]; the extraction lasted 3 h by placing 250 g of dry plant material in a flask with distilled water. The whole was then put to use; the balloon was thus heated, producing vapor charged with volatile products. This vapor condenses in contact with a refrigerant. The condensate was collected in Clevenger, where the separation of the two immiscible phases was carried out: aqueous phase and organic phase. The oils obtained were dehydrated with anhydrous sodium sulfate to eliminate traces of water and stored at 4 °C in the dark before use in shaded glass tubes, hermetically sealed to preserve them from air, light and temperature variations, which are the main agents of alteration of the EO.

The EO yield was calculated using the relationship between mass, oil and moisture, as established by Santos et al. [71].

Yield% = extract (g)/dry matter (g) × 100.

### 3.3. Chemical Characterization of Essential Oils

The chemical composition of the EOs was determined by GC-MS. The samples analyzed on a Thermo Fisher gas chromatograph were coupled to the mass spectrometry system (model GC ULTRA S/N 210729). The column used was a 5% phenylmethyl silicone HP-5 capillary column (30 m × 0.25 mm × thickness 0.25 μm). The temperature was programmed from 50 °C and, after 5 min of initial holding, to 200 °C at 4 °C/min. The carrier gas was N2 (1.8 mL/min); split mode was used with a flow rate of 72.1 mL/min and a ratio of 1/50; the injector and detector temperatures were 250 °C; and the final hold time was 48 min. 1 μL of essential oil was diluted in hexane and injected manually.

### 3.4. Antioxidant Activity

The free radical scavenging activity of essential oils was tested spectrophotometrically by 1,1-diphenyl-2-picryl-hydrazil (DPPH) [72]. A solution of (0.2 mmol/L) DPPH in ethanol was prepared in a 50 mL flask, protected from light and air. In light-protected flasks also solutions of different concentration of each essential oil were prepared subsequently (from 5 to 100 μg/mL), then 0.5 mL of the DPPH solution was added to 2.5 mL of each concentration of EO; the mixture was vortexed and the tubes were placed in the dark at room temperature for 30 min. The optical density (OD) was measured at 517 nm against the blank samples. The same procedure was performed for Trolox, ascorbic acid and BHT of different concentrations. The percentage of DPPH inhibition by the different oil concentrations was calculated and the antioxidant power of EO was represented in Trolox, ascorbic acid and BHT equivalent (mg ET/g EO), (mg EAAs/g EO) and (mg EBHT/g EO). Each test was repeated three times.

### 3.5. Evaluation of Antibacterial Activity

The six microbial strains to be tested (*Staphylococcus aureus*, *Salmonella* sp., *Pseudomonas aeruginosa*, *Escherichia coli*, *Klebsiella pneumonia* and *Streptococcus* sp.) were provided by the Microbiology Department of the Hassan II University Hospital of FES. They were regularly maintained by plating on Muller Hinton agar medium. After 24 h of incubation at 37 °C, bacterial suspensions with an optical density of 1 McFarland were prepared for each strain in 5 mL of sterile water. Evaluation of the antibacterial activity of EOs performed using two different methods.

#### 3.5.1. Disk Diffusion Assay

In Petri dishes, Muller Hinton agar (MH) is poured, after solidification, the agar is inoculated with a freshly prepared microbial suspension. Then a sterile Whatman paper disc is impregnated with 15 μL of the essential oil and is then deposited in the middle of the inoculated agar. Positive controls are also prepared by depositing these time discs of reference antibiotics (Piperacillin 30 µg (PRL 30), Ampicillin 10 µg (AMP 10), Sulphamethoxazole 25 µg (RL25), Penicillin G 5 units (P5), Erythromycin 15 µg (E15) and Streptomycin 10 µg (S10)). The plates were incubated at 37 °C for 24 h. A uniform diffusion of the oil is observed as soon as the impregnated discs are applied; the sensitivity of the strain to the oil is denoted at the end of the incubation period, by measuring the zone of inhibition around the discs in which there was no growth of the microorganisms. The wider the inhibitory zone, the greater the sensitivity of the germ. The larger the zone of inhibition, the more susceptible the germ.

#### 3.5.2. Microdilution in Agar Medium

In a 0.2% agar solution, dilutions of the essential oil are prepared at 1/10, 1/25, 1/50, 1/100, 1/200, 1/300 and 1/500. Then, 1.5 mL of each dilution is added into test tubes, each containing 13.5 mL of sterilized Mueller Hinton medium (MH), and they are cooled to 45 °C. So as to obtain final concentrations of 1/100, 1/250, 1/500, 1/1000, 1/2000, 1/3000 and 1/5000 (*v*/*v*), the mixture was shaken to disperse the oil in the culture medium, and then the tubes were discharged into Petri dishes. Controls containing the culture medium and 0.2% agar solution are prepared in parallel. The minimum inhibitory concentration (MIC) is the lowest concentration of oil that inhibits the growth of microorganisms after 24 h of incubation at 37 °C [73]. The minimum bactericidal concentration of MBC is determined by inoculating a sample from the boxes with no microbial growth on Mueller-Hinton agar; the lowest concentration of oil at which strains do not grow after incubation under the same previous conditions is the MBC. Each trial was repeated three times to minimize experimental error.

### 3.6. Acute Toxicity Study

The acute toxicity study of the three essential oils was evaluated on adult female rats according to the Organization for Economic Cooperation and Development (OECD) guidelines 423 [74,75,76,77,78,79,80,81,82]. Before starting the experiment, the rats were deprived of food for 3–4 h. The weight of the animals was measured to determine the dose to be administered by gavage and to form the 4 groups, each of which consisted of 6 rats; the first group received the dose of 300 mg/kg, the second received 1000 mg/kg, the third received 2000 mg/kg and the fourth group was the control, which received only the table oil. This was applied to all three essential oils. After administration of the EOs, signs of toxicity, mortality and behavioral symptoms (respiration, convulsions, food and water intake) were recorded for each group during the first hour after gavage and at the end of the experiment, i.e., after day 14. The body weight of the animals was also measured every day during the experiment. At the end of the experiment, the 50% lethal dose (LD_50_) was determined according to the protocol described in the OECD guidelines 423 [74].

### 3.7. Statistical Analysis

Data from the microbiological part was analyzed by JMP SAS Pro software (JMP^®^, Version <14>. SAS Institute Inc., Cary, NC, USA). An analysis of variance (ANOVA) was used to evaluate the effect of species, substance (essential oil and antibiotic) and their interaction on the diameter of the inhibition zone. The means of the treatments were compared by Tukey’s HSD (Honestly Significant Difference) test at the 0.05 risk of error.

## 4. Conclusions

The essential oils of three medicinal plants (*C. atlantica*, *C. ambrosioides* and *E. camaldulensis*) collected from several locations in Morocco were examined for their chemical composition, biological antioxidant and anti-microbial capabilities and acute oral toxicity. Different chemical profiles were evident in the examined essences: *C. ambrosioides* essential oil is highly rich in monoterpenes and monoterpenic peroxides, whereas the essential oil of *E. camaldulensis* is richer in monoterpenols and monoterpenic compounds, while *C. atlantica* is richer in sesquiterpenes. The antioxidant assay showed that *E. camaldulensis* EO (99.252 mg EBHT/g EO, 24.167 mg EAAs/g EO and 40.406 mg ET/g EO) has a higher antioxidant power than the other two EOs. The results of the antibacterial effect show that the three essences have an antimicrobial effect on all the strains studied, which is validated by a zone of inhibition with a diameter varying from 7 to 15 mm, and MIC values that vary according to the EO and the strain. The acute oral toxicity test revealed that the LD_50_ of the three EOs was 500 mg/kg. Taken together, these relevant results encourage further research into the factors influencing the biosynthesis and bioactivity of essential oils, as these have gained important applications in the food and pharmaceutical industries. In addition, the results of the biological activities require further testing and studies to find formulations that utilize the antimicrobial properties of these essential oils.

## Figures and Tables

**Table 1:** Chemical composition of the three oils studied.

	Compound	RI	*C. ambrosioides*	Area (%) *E. camaldulensis*	*C. atlantica*
1	α-pinène	936	0.1	4.11	1.08
2	β-myrcène	991	T	-	-
3	Myrcène	993	0.1	-	-
4	α-phellandrène	999	-	2.53	-
5	δ-3-carène	1010	T	-	-
6	3-carène	1011	-	-	1.42
7	p-cymène	1013	12.1	35.11	2.68
8	α-terpinène	1020	53.4	-	-
9	1.8 cinéol	1021	0.5	1.44	-
10	Limonène	1031	0.6	-	6.12
11	γ-terpinène	1051	1.5	3.71	-
12	δ-terpinène	1054	T	-	-
13	2-methylprop-1-enyl-cyclohexa-1,5-dien	1057	-	3.43	-
14	α-terpinolène	1088	-	0.91	-
15	p-cymenène	1090	0.1	-	-
16	Linalool	1099	1.2	0.96	-
17	Terpinen-1-ol	1134	0.7	-	-
18	3-methyl-2-(2-penthenyl)cyclopentanone	1138	-	3.44	-
19	α-terpineol	1175	-	2.67	-
20	Terpinène-4-ol	1176	T	-	-
21	α-campholen aldehyde	1189	-	1.94	-
22	p-cymen-9-ol	1190	0.6	-	-
23	L-linalool	1198	-	11.51	-
24	Ascaridole	1226	17.7	-	-
25	Piperitone	1253	-	10.28	-
26	Thymol	1268	0.1	-	-
27	Carvacrol	1279	7.3	-	-
28	Isoascaridole	1300	2.1	-	-
29	Isoledene	1373	-	-	0.04
30	Longifolène	1387	-	-	3.17
31	Isocaryophyllène	1413	-	-	0.91
32	Himachala-2,4-diène	1424	-	-	0.1
33	Humulène	1437	-	-	1.2
34	γ- himachalène	1476	-	-	15.54
35	Himachalène	1499	-	-	1.16
36	β-himachalène	1514	-	-	54.21
37	δ -cadinène	1524	-	-	3.24
38	Himachalène oxide	1564	-	-	6.23
39	Limonène Oxide	1586	1.3	-	-
40	Globulol	1590	-	6.06	-
41	ɣ-eudesmol	1634	-	11.9	-
42	β -sinensal	1697	-	-	0.01
	Total identified %		99.4	100	97.11

RI: retention index, T: trace components.

**Table 2:** Antioxidant activity of EOs, values are given as means of three replicates ± SD.

EOs Samples	DPPH (mg BHTE of EO)	DPPH (mg AAE of EO)	DPPH (mg TE of EO)
*Cedrus atlantica*	53.928 ± 0.01	17.713 ± 0.40	26.208 ± 0.12
*Chenopodium ambrosioides*	72.783 ± 0.19	18.999 ± 0.27	30.816 ± 0.40
*Eucalyptus camaldulensis*	99.252 ± 0.01	24.167 ± 0.76	40.406 ± 0.51

BHTE: hydroxytoluene equivalent, AAE: ascorbic acid equivalent and TE: Trolox equivalent.

**Table 3:** ANOVA results to test the diameter of the zone of inhibition between the substances (essential oil and antibiotics) and the bacterial species.

Source of Variation	DF	Sum of Squares	Mean Square	F Ratio	*p*-Value
Bacterial species	5	3809.51	761.90	272.835	<0.0001 *
Substances	11	9595.01	872.27	312.358	<0.0001 *
Bacterial species × substances	55	5947.92	108.14	38.726	<0.0001 *
Model	71	19,314.56	272.04	97.415	<0.0001 *
Error	143	399.33	2.79		
C. Total	214	19,713.89			

*: significant at 0.1% level; DF: degree of freedom.

**Table 4:** MIC and MBC of essential oils.

Souches Bactériennes	*C. atlantica*		*E. camaldulensis*		*C. ambrosioides*	
	CMI	CMB	CMI	CMB	CMI	CMB
*Salmonella* sp.	1/1000	1/1000	Nd	Nd	1/500	1/500
*S. aureus*	1/2000	1/2000	Nd	Nd	1/1000	1/500
*E. coli*	1/1000	1/1000	Nd	Nd	1/1000	1/1000
*K. pneumoniae*	1/2000	1/2000	Nd	Nd	1/500	1/500
*Ps. aeruginosa*	Nd	Nd	Nd	Nd	Nd	Nd
*Strypto* sp.	1/1000	1/500	1/250	1/250	1/1000	1/500

Nd: not determined.

**Table 5:** Results of acute oral toxicity tests of oils with different doses.

Essential Oils	Number of Rats Per Batch	Doses mg/Kg	Mortality	Signs of Toxicity	LD_50_	Category GHS (mg/Kg)
*Eucalyptus camaldulensis*	6	300	0	−	500	4
	6	1000	1	+		
	6	2000	3	+		
*Cedrus atlantica*	6	300	0	−	500	4
	6	1000	3	+		
	6	2000	3	+		
*Chenopodium ambrosioides*	6	300	1	+	500	4
	6	1000	3	+		
	6	2000	3	+		

(LD_50_): Lethal dose 50; GHS: harmonized classification system.

**Table 6:** Place of harvest and plant parts used for extraction.

Plants	Place of Harvest	Parts of the Plant Used
*Cedrus atlantica*	Azrou (Middle Eastern Atlas)	Branch and needle
*Chenopodium ambrosioides*	Region of Safi (central Morocco)	Leaves, flowers, stems and seeds
*Eucalyptus camaldulensis*	Maâmora Forest (north west)	Leaves and flowers

## Data Availability

The datasets generated during and/or analyzed during the current study are available from the corresponding author on reasonable request.

## Sample Availability

Samples of the compounds are available from the authors.
